# Janus Faced HMGB1 and Post-Aneurysmal Subarachnoid Hemorrhage (aSAH) Inflammation

**DOI:** 10.3390/ijms231911216

**Published:** 2022-09-23

**Authors:** Shafqat Rasul Chaudhry, Sumaira Shafique, Saba Sajjad, Daniel Hänggi, Sajjad Muhammad

**Affiliations:** 1 Department of Pharmacy, Obaid Noor Institute of Medical Sciences (ONIMS), Mianwali 42200, Pakistan; 2 Department of Biochemistry and Biotechnology, University of Veterinary and Animal Sciences (UVAS), Lahore 54000, Pakistan; 3 Department of Oral-, Maxillofacial and Facial Plastic Surgery, University Hospital Düsseldorf, 40225 Düsseldorf, Germany; 4 Department of Neurosurgery, Faculty of Medicine, University Hospital Düsseldorf, Heinrich-Heine University of Düsseldorf, Moorenstr. 5, 40225 Düsseldorf, Germany; 5 Department of Neurosurgery, University of Helsinki and Helsinki University Hospital, 00029 Helsinki, Finland

**Keywords:** SAH, CVS, serum biomarkers, stroke, inflammation, cysteine, pro-resolving, anti-inflammatory, DAMPs

## Abstract

Aneurysmal subarachnoid hemorrhage (aSAH), resulting majorly from the rupture of intracranial aneurysms, is a potentially devastating disease with high morbidity and mortality. The bleeding aneurysms can be successfully secured; however, the toxic and mechanical impact of the blood extravasation into the subarachnoid space damages the brain cells leading to the release of different damage-associated molecular pattern molecules (DAMPs). DAMPs upregulate the inflammation after binding their cognate receptors on the immune cells and underlies the early and delayed brain injury after aSAH. Moreover, these molecules are also associated with different post-aSAH complications, which lead to poor clinical outcomes. Among these DAMPs, HMGB1 represents a prototypical protein DAMP that has been well characterized for its proinflammatory role after aSAH and during different post-aSAH complications. However, recent investigations have uncovered yet another face of HMGB1, which is involved in the promotion of brain tissue remodeling, neurovascular repair, and anti-inflammatory effects after SAH. These different faces rely on different redox states of HMGB1 over the course of time after SAH. Elucidation of the dynamics of these redox states of HMGB1 has high biomarker as well as therapeutic potential. This review mainly highlights these recent findings along with the conventionally described normal role of HMGB1 as a nuclear protein and as a proinflammatory molecule during disease (aSAH).

## 1. Introduction

Subarachnoid hemorrhage (SAH) is a cerebrovascular disease that is characterized by bleeding into the subarachnoid space and sometimes may also accompany intraventricular or intracerebral bleeding [1]. The majority of SAH cases are a consequence of the rupture of an intracranial aneurysm [2,3]. Intracranial aneurysms often occur at the arterial bifurcation sites owing to chronic shear stress and chronic inflammation leading to the formation of weakened bulging lesions in the arterial brain vessels [4,5]. Almost 3–5% of the population harbors these intracranial aneurysms, with a slight prevalence in females [5]. Rupture of intracranial aneurysms introduces blood into the subarachnoid space at high pressure, leading to mechanical damage as well as interference with cerebral perfusion due to elevated intracranial pressure resulting in transient global ischemia. Moreover, the toxic effects of the blood and its derivatives cause stress and damage to the brain cells [1,6]. The bleeding aneurysms can be repaired through endovascular coiling or neurosurgical clipping; however, still, a large proportion of the patients deteriorate and attain poor clinical outcomes [7]. The events occurring over 72 h after aSAH cause early brain injury (EBI), which is characterized by highly upregulated local and systemic inflammation. Further, over the course of post-aSAH sequel, aSAH patients develop life-threatening complications. These include cerebral vasospasm (CVS), acute and chronic hydrocephalus, seizures, delayed cerebral ischemia (DCI), cortical spreading depression (CSD), and local/systemic infections [8]. These complications and ongoing inflammation probably contribute to poor clinical outcomes.

aSAH is characterized by a robust sterile inflammatory response immediately after the subarachnoidal bleed. Several damage-associated molecular pattern molecules (DAMPs) are liberated upon injury of the brain cells from different intracellular compartments and have the capability to activate immune cells through the ligation of their cognizant receptors (pattern recognition receptors (PRRs)) [7]. Over the recent years, an ever-expanding list of DAMPs have been discovered with varying natures, e.g., protein DAMPs such as high mobility group box-1 (HMGB1), peroxiredoxins, S100β; nucleic acid-based DAMPs such as nuclear and mitochondrial DNA, and RNA, ATP; extracellular matrix proteins and other components such as hyaluronan, tenascin C; monosodium urate crystals and cholesterol stones; hemoglobin and its derivatives, etc. These diverse molecules with different chemical natures bind to different PRRs belonging to different families and are expressed extracellularly on the cell surface as well as intracellularly on immune and other cells [7]. The accumulating evidence shows that several DAMPs molecules are upregulated after aSAH both at the CNS level and peripherally [7]. Along with these DAMPs, the expression of several PRRs is also upregulated after aSAH [7]. These DAMPs not only serve as the initiators of the acute inflammation, rather they are persistently upregulated to sustain the inflammation after aSAH and during post-aSAH complications [7].

High mobility group box 1 (HMGB1) is one of the non-histone DNA binding proteins that is ubiquitously expressed in the nucleus of all eukaryotic cells. Its name is derived from the fact that it showed high electrophoretic mobility on a polyacrylamide gel and was discovered in 1973 [9]. It is composed of 215 amino acids that consist of two boxes, i.e., HMG Box A (1–79 amino acids) and B (89–162 amino acids), and an acidic tail containing aspartic acid and glutamic acid residues (186–215 amino acids) [9]. HMGB1 is involved in several vital functions within the nucleus, such as DNA repair, transcription, telomere maintenance, and genome stability [9]. The tail at the C-terminus is involved in the spatial arrangement of the Box A and Box B of HMGB1 and controls the DNA binding specificity of HMGB1 [10]. However, in contrast to these normal functions, HMGB1 can upregulate the inflammatory response extraordinarily due to its DAMP nature once released extracellularly upon cellular injury [11]. HMGB1 binds several pattern recognition receptors (PRRs). The most important of them are TLR-2, TLR-4, and RAGE (receptors for advanced glycation end products) on immune cells to upregulate inflammation [11]. 

A great body of evidence highlights the inflammatory role of HMGB1 in several different types of diseases (Table 1). HMGB1 has been shown to be implicated in the pathophysiology of sepsis, meningitis, pneumonia, systemic vasculitis, atherosclerosis, abdominal aortic aneurysms, cardiac, kidney, respiratory and gastrointestinal diseases, hepatic damage, autoimmune disorders, metabolic disorders, and several types of neoplastic disorders such as renal cell carcinoma, lung cancer, pancreatic cancer, gastric cancer, colorectal cancer, hepatocellular carcinoma, etc. [12].

## 2. HMGB1 and Neurological Disorders

As HMGB1 is ubiquitously expressed in cells and is an important weapon in the immune arsenal, therefore, it is not surprising that it is involved in the pathophysiology of several diseases [11]. In addition to the diseases mentioned above, the role of HMGB1 has been well characterized in the pathobiology of different nervous system disorders. HMGB1 expression has been investigated in Alzheimer’s disease, amyotrophic lateral sclerosis, Huntington’s disease, Parkinson’s disease, multiple sclerosis, neuromyelitis optica, neuropathic pain, epilepsy, stroke, intracerebral hemorrhage, and traumatic brain injury (Table 1) [12,13,14,15]. The significance of HMGB1 can be envisaged by the fact that neuronal expression of HMGB1 declines during aging, whereas its expression in astrocytes is upregulated, and reduced neuronal HMGB1 correlates with the accumulation of double-stranded DNA breaks [16].

## 3. HMGB1 and Ischemic Stroke

Ischemic stroke is one of the leading causes of death and morbidity worldwide. The pathophysiology of stroke is complex, and inflammation plays a critical role in ischemic brain damage [17]. HMGB1, like in other inflammatory diseases, plays a central role in inflammation after ischemic stroke [18]. HMGB1 has been shown to be translocated in the cytosol after ischemic stroke and is released extracellularly, which is implicated in the blood–brain barrier (BBB) impairment, neuroimmune activation, and neuronal demise [17]. HMGB1 undergoes modifications such as acetylation or phosphorylation in microglia and astrocytes, which renders the interaction between HMGB1 and DNA weak and enables it to translocate to the cytoplasm, and, finally, it is actively secreted extracellularly [19]. HMGB1 has been shown to be detected in the serum of ischemic stroke patients and in the brain tissue of mice after experimental ischemic stroke as early as two hours after stroke [20]. Several studies have documented excessively raised levels of HMGB1 in both cerebrospinal fluid (CSF) and peripheral blood plasma/serum of ischemic stroke patients [17]. The studies focusing on investigating the levels of HMGB1 have found that HMGB1 levels peak early during the acute stage after ischemic stroke, consistent with the passive release of HMGB1 from necrotic neurons and later on 6–7 days after ischemic stroke, reflecting the actively released HMGB1 from the activated microglia, macrophages, endothelial cells and astrocytes [21]. However, highly raised HMGB1 levels in the blood of ischemic stroke patients have been traced up to one month after stroke [22]. Interestingly, a fully reduced form of HMGB1 has been found in the brain and serum early after experimental cerebral ischemia; however, the oxidized disulfide form of HMGB1 with a cytokine-inducing capability dominates after 24 h of middle cerebral artery occlusion (MCAO) [17]. 

HMGB1, upon release after ischemic stroke, has been shown to bind several receptors, such as TLRs and RAGE, on immune cells to extend the initial damage caused by ischemic insult [14]. HMGB1 has been found to be associated with the release of pro-inflammatory cytokines such as IL-1β, TNF-α, IFN-γ, and IL-6, and the activation of NF-κB and inflammasome activation after experimental ischemic stroke. Moreover, HMGB1 has been shown to be associated with larger infarct sizes and BBB disruption, as all of these changes were improved upon antagonizing HMGB1 after experimental cerebral ischemia [14,17,20]. 

Interestingly, as the late phase of the inflammation is generally characterized by the upregulation of the pro-resolving and tissue remodeling mechanisms, similarly, HMGB1 in the late phase of stroke is suggested to promote the regeneration of neuronal cells, blood vessels remodeling, and recovery of neurological function [23]. Consequently, HMGB1 released by astrocytes in the late phase of stroke promotes the viability and migration of endothelial progenitor cells leading to repair of the neurovascular unit, peri-infarct angiogenesis, and improvement in neurological function [24]. These lines of evidence suggest that HMGB1 plays a dual role in stroke pathophysiology, the early being damaging, while the later on pro-resolving and facilitating the remodeling of the brain tissue.

## 4. HMGB1 and Subarachnoid Hemorrhage

Subarachnoid hemorrhage is a highly fatal and morbid disease and results in the majority of cases due to the rupture of intracranial aneurysms. A huge body of evidence highlights the critical role of inflammation in the post-SAH sequelae. Several studies have shown that HMGB1 plays an important role in post-SAH inflammation (Table 1) [7]. HMGB1 levels have been found to increase significantly in the CSF of the patients after aSAH [25]. HMGB1 increase in the CSF of aSAH patients is accompanied by a parallel increase in the CSF levels of different proinflammatory cytokines such as TNF-α, IL-6, and IL-8, suggesting an inflammatory role of HMGB1 [25]. HMGB1 CSF levels in aSAH patients are associated with the severity of aSAH and poor neurological functional scores [26,27]. Further, raised CSF HMGB1 levels show an association with poor clinical outcomes and disability [26,27]. HMGB1 levels also increase in systemic circulation after aSAH [28,29]. Interestingly, CSF HMGB1 and systemic HMGB1 levels have been found to be associated with the development of acute hydrocephalus and cerebral vasospasm (CVS), respectively [27,28,29]. Chaudhry et al. have found an early and sustained elevation of serum HMGB1 levels in aSAH patients who developed CVS over a period of two weeks [29]. CVS is one of the most frequent and feared complications after aSAH that contributes majorly to the development of delayed cerebral ischemia (DCI) [1,7]. Furthermore, genetic polymorphism of HMGB1 that may lead to enhanced expression of HMGB1 has been shown to be associated with delayed cerebral ischemia (DCI), which is one of the leading causes of the delayed deterioration in aSAH patients after the sentinel bleed [30]. Recently, Hemmer et al. have shown in a larger cohort of aSAH patients that admission serum HMGB1 levels and angiographic vasospasm independently predict the development of DCI [31]. DCI increases the morbidity and increases the risk of poor clinical outcomes by two-folds in aSAH patients [1]. 

## 5. HMGB1 and Experimental Subarachnoid Hemorrhage

Like the clinical studies advocating the important role of HMGB1 in post-aSAH inflammation, several studies conducted on different animal models mimicking SAH have successfully translated the inflammatory role of HMGB1, providing deeper insights into the understanding of the involvement of the cognate receptors and the implication of substream intracellular pathways that keep up the process of inflammation. Induction of SAH has been shown to increase the expression of HMGB1, and HMGB1 was majorly expressed in the cytosol of microglial cells/macrophages as the cells that showed the expression of HMGB1 also expressed Iba1 (which is a marker of microglia/macrophages) [32]. Another study showed that HMGB1 is released from neurons as early as 2 h after experimental subarachnoid hemorrhage. However, HMGB1 cytoplasmic translocation was seen mainly in the neurons and also in some microglia after SAH induction [33]. This time profile for the early release of HMGB1 after SAH coincides with that seen during ischemic stroke, as described above [20]. Further, several investigations have shown that HMGB1 is associated with an upregulation of the inflammatory response characterized by the release of proinflammatory cytokines, activation of the inflammatory intracellular pathways leading to NF-κB activation, BBB disruption and brain edema, apoptosis, necroptosis, and poor neurological functional scores [34]. Intriguingly, as mentioned above, CVS is considered a frequent complication and major contributor to DCI after aSAH; therefore, several studies have experimentally investigated the impact of HMGB1 on CVS development by evaluating the expression in basilar arteries. The arteries harvested from the experimental animals showing vasospasm exhibited the upregulated expression of HMGB1 along with other inflammatory markers [7,34]. These lines of evidence confirm the involvement of HMGB1 in post-aSAH inflammation and during various post-aSAH complications.

## 6. Anti-HMGB1 Therapies and Subarachnoid Hemorrhage

Glycyrrhizic acid is an inhibitor of HMGB1, and its application in an in-vitro model of SAH has been shown to antagonize the inflammatory effects of HMGB1. During this study, glycyrrhizic acid application prevented the upregulated release of IL-1β from mixed glial cells subjected to the medium obtained after the application of hemoglobin (Hb) to neuronal culture in-vitro [33]. Several other natural products such as purpurogallin (a natural phenol) and 4′-*O*-β-d-glucosyl-5-*O*-methylvisamminol (4OGOMV), rhinacanthin-C (an extract from *Rhinacanthus nasutus*), resveratrol, glycyrrhizin, and berberine have been shown to downregulate the expression of HMGB1 in different models of SAH [34,35]. All these potential treatments afforded several beneficial effects due to the antagonism of HMGB1, such as the downregulation of proinflammatory cytokine expression, downregulation of the intracellular inflammatory pathways, reduced brain edema and neuronal apoptosis, reversal of the cerebral vasospasm, and improvement in the neurological function and outcome [7,34]. Besides these natural products, anti-HMGB1 monoclonal antibody administration ameliorates the HMGB1-induced inflammation in a model of SAH, further inhibits the development of CVS, and protects from brain injury [36]. Interestingly, the administration of GSK’872, an inhibitor of RIPK-3, prevented the cytosolic translocation and expression of HMGB1 and necroptosis after experimental SAH and reduced BBB disruption, and improved neurological outcomes [37]. Even exosomes derived from the mesenchymal stem cells and the use of soluble decoy form of RAGE have been shown to protect against the damage due to HMGB1-induced inflammation after experimental SAH [38,39]. These lines of evidence clearly demonstrate an early inflammatory role of the HMGB1 in post-SAH sequelae and the therapeutic value of early antagonism of HMGB1.

## 7. Pro-Resolving and Protective Effects of HMGB1 and Subarachnoid Hemorrhage

Neurovascular remodeling continues days to weeks after stroke and involves the cross-talk of neurons, glia, endothelial cells, and extracellular matrix components [40]. Reactive astrocytes release numerous growth factors such as nerve growth factor (NGF), vascular endothelial growth factor (VEGF), brain-derived neurotrophic factor (BDNF), and other factors after stroke, which participate in brain tissue remodeling, peri-infarct angiogenesis and repair of the neurovascular units to improve the neurological functional outcomes after brain injury [24,41,42]. As already mentioned above, HMGB1 unveils its second face during the late phase of the stroke involving brain tissue remodeling and repair. HMGB1 secreted by the astrocytes during the late phase of stroke promotes the function of the endothelial progenitor cells, which support the repair of the neurovascular unit and angiogenesis near the infarct territories [24]. Moreover, HMGB1 has already been shown to promote endothelial activation and sprouting [43]. Further, HMGB1 promotes neurogenesis after intracerebral hemorrhage (ICH) in a RAGE-dependent manner [44].

Intriguingly, a study investigating the effects of HMGB1 during the late phase of SAH unveiled the neuroprotective and pro-resolving effects of HMGB1 as opposed to conventional proinflammatory DAMPs-based effects during the early brain injury after SAH [42]. Tian and co-authors utilized ethyl pyruvate and glycyrrhizic acid to inhibit HMGB1, and FPS-ZM1 to inhibit the expression of RAGE. Consequently, authors were able to delineate the reduced expression of HMGB1 and RAGE upon inhibitors administration; however, it was associated with reduced levels of the different neurogenic growth factors such as NGF, VEGF, and BDNF and also reduction in the cortical neurogenesis as reflected by a decrease in BrDU and DXC-positive neurons [42]. It is interesting to note that different isoforms of HMGB1 exist with distinct redox states of the three cysteine residues (C23, C45, and C106) in the box B of the HMGB1 molecule, and these isoforms have been characterized to function differently over the course of the disease [29]. The cysteine residues in reduced and thiolated form have chemotactic properties, whereas disulfide-linked C23-S–S-C45 possess proinflammatory cytokine-like activity, and the fully oxidized and sulfonated form is inert and found during the resolution of inflammation [29]. Therefore, in the study of Tian and colleagues, two modified forms of HMGB1 at C106 were used, i.e., one isoform that was oxidized at the C106 position and the other in which C106 was in the reduced state [42]. So, when the reduced form of HMGB1, which retains the cytokine-inducing capability, was used, it aggravated the brain damage. However, utilization of the oxidized form of HMGB1 with no cytokine stimulating potential led to a decrease in the production of TNF-α and promoted the recovery of the brain through the upregulation of the neurotrophic factors [42]. Furthermore, these effects were also abolished upon antagonism of RAGE, suggesting that the oxidized form of HMGB1 mediates neuroprotective, anti-inflammatory, and pro-resolving effects through RAGE [42].

SAH is characterized by a robust oxidative stress response as evidenced by the progressive decline in glutathione and other antioxidant agents along with an early increase in lipid peroxidation products, which decline with the passage of time in the peripheral circulation of aSAH patients [45]. Hemoglobin and its degradation products liberated from the extravasated SAH blood upregulates the generation of reactive oxygen species (ROS), leading to oxidation of cellular components, and ROS also triggers several subcellular inflammatory pathways [46]. Microglia, the resident immune cells of the CNS, polarize to M1 proinflammatory phenotype during an early phase of SAH and then dynamically shift into M2 anti-inflammatory and tissue reparative phenotype [47]. Interestingly, M1-microglia upregulate the generation of ROS and induce the activation of NF-κB-mediated proinflammatory genes expression early in the inflammatory response. Later on, microglia polarize to an M2 phenotype that is characterized by the upregulation of antioxidant machinery during the late phase of an inflammatory response and supports tissue repair and resolution of inflammation [48]. Interestingly, it is known that under mild oxidative conditions, C23 and C45 are cross linked through disulfide bridge and exert a potent pro-inflammatory response [49]. Consequently, it has been shown that this disulfide form of HMGB1 is associated with priming and neuroinflammatory response in microglia [50]. Intriguingly, the disulfide isoform of HMGB1 has also been shown to reduce the proliferation of oligodendrocyte precursor cells [51]. However, the redox-dependent dual roles of HMGB1 in connection to the different polarized states of microglia during early and delayed brain injury after SAH are still to be investigated.

The above-mentioned evidence clearly shows the second face of HMGB1, which is evident during the late phase of SAH. Consequently, it is intimidating to further explore the effects of different anti-HMGB1 therapies during the delayed brain injury phase after aSAH. Furthermore, there is also a need to validate the dynamics of different isoforms of HMGB1 over the phases of EBI and DBI and their contribution to different post-aSAH complications and neuroinflammation. Interestingly, it opens new avenues for the development of isoform-selective anti-HMGB1 therapies that may be utilized to curb the damaging effects of inflammation and boost the reparative anti-inflammatory effects.

## 8. Conclusions

HMGB1 is a non-histone nuclear protein that binds chromatin and facilitates transcription under normal conditions. However, extracellular HMGB1 release from necrotic cells acts as a DAMP and activates immune response leading to inflammation. In addition to these widely known conventional dual roles of HMGB1 (homeostatic or DAMP roles), HMGB1 displays yet another pro-resolving, anti-inflammatory and neuroprotective role during the late phase of SAH, which is dependent on the oxidized isoform of HMGB1. The disulfide isoform of HMGB1 promotes proinflammatory responses, whereas the fully reduced isoform displays chemotactic properties. It would be interesting to delineate the oxidative states in relation to the dynamics of HMGB1 isoforms and microglial polarization during the early and delayed brain injury phases after SAH. Elucidation of the dynamics of these isoforms, making HMGB1 Janus faced, will further unleash the biomarker and therapeutic potential of HMGB1. For instance, antagonism of the disulfide form of HMGB1 is warranted during the early brain injury phase after aSAH. Further, determining the dynamics of these isoforms of HMGB1 in association with different post-aSAH complications will serve diagnostic and prognostic purposes.

## Figures and Tables

**Table 1:** List of different diseases in which HMGB1 levels are altered.

Sr. No.	Disease Condition	Study Type	No. of Patients	Sample Measured	Time Duration of Measurement	HMGB1 Status	Other Biomarkers	Reference
1.	Sepsis and septic shock	Observational cohort study	Derivation cohort: controls = 46, sepsis = 58, septic shock = 84 validation cohort: sepsis = 24, septic shock = 53	Plasma	One time within 48 h	↑sed	RIPK3, MLKL	[52]
2.	Coronary artery disease (CAD)	Observational study	CAD = 98, controls = 30	Serum	Day after admission	↑sed	High sensitivity (hs) CRP, cardiac troponin I	[53]
3.	ST segment elevation myocardial infarction (STEMI) and mortality	Observational study	STEMI patients = 141, healthy controls (HCS) = 42	Plasma	At admission	↑sed after STEMI and doubled in patients who died	Troponin I, creatine kinase myocardium	[54]
4.	Community-acquired pneumonia (CAP)	Subjects drawn from larger genetic and inflammatory markers of sepsis (GenIMS) study	CAP patients = 122, healthy controls = 38	Plasma	After enrolment, daily for 1st week, then weekly until discharge	↑sed	IL-6, IL-10, TNF-α	[55]
5.	Chronic kidney disease (CKD)	Cross-sectional study	CKD = 177, healthy controls = 48	Serum	After overnight fast	↑sed	hs-CRP, TNF-α, IL-6, Hb, HbA_1c_	[56]
6.	Juvenile idiopathic arthritis (JIA)	Prospective longitudinal study	JIA children = 64, reactive arthritis = 9, HC = 15	Serum	1st visit and at 1st, 3rd, and 6th month follow-up	↑sed	CRP, neutrophils, ferritin, ESR	[57]
7.	Systemic lupus erythematosus (SLE)	Observational study	SLE patients = 70, HC = 35	Serum	At outpatient clinic visit	↑sed in quiescent patients, ↓sed in patients with active SLE	CRP, C3, C4, creatinine, anti-HMGB1, anti-dsDNA	[58]
8.	Type 1 diabetes mellitus (DM)	Case control observational study	Type 1 DM patients = 96, HC = 40	Serum	Within 24 h of diagnosis of type 1 DM	↑sed	CRP, WBCs, glucose, HBA_1C_, β cell autoantibodies	[59]
9.	Type 2 diabetes mellitus (T2DM)	Cross-sectional study	T2DM = 76, normal glucose tolerant (NGT) = 79	Plasma	At outpatient visit	↑sed	IL-6, glucose, insulin, HBA_1C_	[60]
10.	Epilepsy	Case control study	Epilepsy patients = 105, HC = 100	Serum	Within 12 h after seizure	↑sed	TLR-4	[61]
11.	Alzheimer’s disease (AD)	Case control study	AD patients = 24, controls = 12	Serum	Upon recruitment and diagnosis	↑sed	s100β, Aβ, sRAGE, sThrombomodulin antigen	[62]
12.	Parkinson’s disease (PD)	Observational study	PD patients = 120, HC = 100	Serum	After 12 h of fasting after admission	↑sed	TLR-4, MyD88, NFκB, TNF-α	[63]
13.	Multiple sclerosis (MS)	Cross-sectional study	MS patients = 96, HC = 34	Serum	On recruitment	↑sed	-	[64]
14.	Ischemic stroke (IS)	Observational study	IS patients = 183, HC = 16	Serum	On admission and on day 7	↑sed	hs-CRP	[65]
15.	Intracerebral hemorrhage (ICH)	Prospective observational study	ICH patients = 65, HC = 41	Serum	On admission	↑sed	TNF-α, IL-6	[66]
16.	Aneurysmal subarachnoid hemorrhage (aSAH)	Observational study	aSAH patients = 39, Controls = 13	Cerebrospinal fluid (CSF)	Day 3, 7, and 14	↑sed	TNF-α, IL-8, IL-6	[25]
17.	Aneurysmal subarachnoid hemorrhage (aSAH)	Observational study	aSAH patients = 9, Controls = 7	CSF	After admission	↑sed	-	[26]
18.	Aneurysmal subarachnoid hemorrhage (aSAH)	Observational study	aSAH patients = 10, Controls = 8	CSF	Day 1, 5, and 10	↑sed	CRP, fibrinogen, WBCs	[27]
19.	Aneurysmal subarachnoid hemorrhage (aSAH)	Observational study	aSAH patients = 40, Controls = 5	CSF	Post hemorrhage day 7	↑sed	Glucose, lactic acid, protein, WBCs	[39]
20.	Aneurysmal subarachnoid hemorrhage (aSAH)	Observational study	aSAH patients = 303, HC = 150	Plasma	On admission within 48 h	↑sed	-	[28]
21.	Aneurysmal subarachnoid hemorrhage (aSAH)	Retrospective observational study	aSAH patients = 53, controls = 28	Serum	Day 1, 3, 5, 7, 9, 11, and 13	↑sed	IL-6, WBCs	[29]
22.	Aneurysmal subarachnoid hemorrhage (aSAH)	Prospective single-blinded observational study	aSAH patients = 83	Serum	Day 0, 4, 8, and 12	↑sed on admission in aSAH patients with Delayed cerebral ischemia	CRP, WBCs, platelets	[31]

↑ Increased.

## References

[1] Macdonald R.L. (2014). Delayed neurological deterioration after subarachnoid haemorrhage. Nat. Rev. Neurol..

[2] Macdonald R.L., Schweizer T.A. (2017). Spontaneous subarachnoid haemorrhage. Lancet.

[3] van Gijn J., Kerr R.S., Rinkel G.J.E. (2007). Subarachnoid haemorrhage. Lancet.

[4] Lawton M.T., Vates G.E. (2017). Subarachnoid Hemorrhage. N. Engl. J. Med..

[5] Etminan N., Rinkel G.J. (2016). Unruptured intracranial aneurysms: Development, rupture and preventive management. Nat. Rev. Neurol..

[6] Chaudhry S.R., Frede S., Seifert G., Kinfe T.M., Niemelä M., Lamprecht A., Muhammad S. (2019). Temporal profile of serum mitochondrial DNA (mtDNA) in patients with aneurysmal subarachnoid hemorrhage (aSAH). Mitochondrion.

[7] Chaudhry S.R., Hafez A., Rezai Jahromi B., Kinfe T.M., Lamprecht A., Niemela M., Muhammad S. (2018). Role of damage associated molecular pattern molecules (DAMPs) in aneurysmal subarachnoid hemorrhage (aSAH). Int. J. Mol. Sci..

[8] Suarez J.I., Tarr R.W., Selman W.R. (2006). Aneurysmal Subarachnoid Hemorrhage. N. Engl. J. Med..

[9] He S.-J., Cheng J., Feng X., Yu Y., Tian L., Huang Q. (2017). The dual role and therapeutic potential of high-mobility group box 1 in cancer. Oncotarget.

[10] Musumeci D., Roviello G.N., Montesarchio D. (2014). An overview on HMGB1 inhibitors as potential therapeutic agents in HMGB1-related pathologies. Pharmacol. Ther..

[11] Lotze M.T., Tracey K.J. (2005). High-mobility group box 1 protein (HMGB1): Nuclear weapon in the immune arsenal. Nat. Rev. Immunol..

[12] Kang R., Chen R., Zhang Q., Hou W., Wu S., Cao L., Huang J., Yu Y., Fan X.-G., Yan Z. (2014). HMGB1 in health and disease. Mol. Asp. Med..

[13] Lei C., Geng J., Zhong L. (2020). The association between plasma HMGB1 and sRAGE and clinical outcome in intracerebral hemorrhage. J. Neuroimmunol..

[14] Muhammad S., Barakat W., Stoyanov S., Murikinati S., Yang H., Tracey K.J., Bendszus M., Rossetti G., Nawroth P.P., Bierhaus A. (2008). The HMGB1 receptor RAGE mediates ischemic brain damage. J. Neurosci. Off. J. Soc. Neurosci..

[15] Jassam Y.N., Izzy S., Whalen M., McGavern D.B., El Khoury J. (2017). Neuroimmunology of traumatic brain injury: Time for a paradigm shift. Neuron.

[16] Enokido Y., Yoshitake A., Ito H., Okazawa H. (2008). Age-dependent change of HMGB1 and DNA double-strand break accumulation in mouse brain. Biochem. Biophys. Res. Commun..

[17] Ye Y., Zeng Z., Jin T., Zhang H., Xiong X., Gu L. (2019). The role of high mobility group box 1 in ischemic stroke. Front. Cell Neurosci..

[18] Huang J.M., Hu J., Chen N., Hu M.L. (2013). Relationship between plasma high-mobility group box-1 levels and clinical outcomes of ischemic stroke. J. Crit. Care.

[19] Singh V., Roth S., Veltkamp R., Liesz A. (2016). HMGB1 as a key mediator of immune mechanisms in ischemic stroke. Antioxid. Redox Signal..

[20] Liesz A., Dalpke A., Mracsko E., Antoine D.J., Roth S., Zhou W., Yang H., Na S.Y., Akhisaroglu M., Fleming T. (2015). DAMP signaling is a key pathway inducing immune modulation after brain injury. J. Neurosci. Off. J. Soc. Neurosci..

[21] Kim J.B., Lim C.M., Yu Y.M., Lee J.K. (2008). Induction and subcellular localization of high-mobility group box-1 (HMGB1) in the postischemic rat brain. J. Neurosci. Res..

[22] Schulze J., Zierath D., Tanzi P., Cain K., Shibata D., Dressel A., Becker K. (2013). Severe stroke induces long-lasting alterations of high-mobility group box 1. Stroke.

[23] Le K., Mo S., Lu X., Idriss Ali A., Yu D., Guo Y. (2018). Association of circulating blood HMGB1 levels with ischemic stroke: A systematic review and meta-analysis. Neurol. Res..

[24] Hayakawa K., Pham L.D., Katusic Z.S., Arai K., Lo E.H. (2012). Astrocytic high-mobility group box 1 promotes endothelial progenitor cell-mediated neurovascular remodeling during stroke recovery. Proc. Natl. Acad. Sci. USA.

[25] Nakahara T., Tsuruta R., Kaneko T., Yamashita S., Fujita M., Kasaoka S., Hashiguchi T., Suzuki M., Maruyama I., Maekawa T. (2009). High-mobility group box 1 protein in CSF of patients with subarachnoid hemorrhage. Neurocrit. Care.

[26] King M.D., Laird M.D., Sangeetha S.R., Youssef P., Shakir B., Vender J.R., Alleyne C.H., Dhandapani K.M. (2010). Elucidating novel mechanisms of brain injury following subarachnoid hemorrhage: An emerging role for neuroproteomics. Neurosurg. Focus.

[27] Sokol B., Wozniak A., Jankowski R., Jurga S., Wasik N., Shahid H., Grzeskowiak B. (2015). HMGB1 level in cerebrospinal fluid as a marker of treatment outcome in patients with acute hydrocephalus following aneurysmal subarachnoid hemorrhage. J. Stroke Cerebrovasc. Dis. Off. J. Natl. Stroke Assoc..

[28] Zhu X.-D., Chen J.-S., Zhou F., Liu Q.-C., Chen G., Zhang J.-M. (2012). Relationship between plasma high mobility group box-1 protein levels and clinical outcomes of aneurysmal subarachnoid hemorrhage. J. Neuroinflamm..

[29] Chaudhry S.R., Guresir A., Stoffel-Wagner B., Fimmers R., Kinfe T.M., Dietrich D., Lamprecht A., Vatter H., Guresir E., Muhammad S. (2018). Systemic high-mobility group box-1: A novel predictive biomarker for cerebral vasospasm in aneurysmal subarachnoid hemorrhage. Crit. Care Med..

[30] Hendrix P., Foreman P.M., Harrigan M.R., Fisher W.S.R., Vyas N.A., Lipsky R.H., Lin M., Walters B.C., Tubbs R.S., Shoja M.M. (2017). Impact of high-mobility group box 1 polymorphism on delayed cerebral ischemia after aneurysmal subarachnoid hemorrhage. World Neurosurg..

[31] Hemmer S., Senger S., Griessenauer C.J., Simgen A., Oertel J., Geisel J., Hendrix P. (2022). Admission serum high mobility group box 1 (HMGB1) protein predicts delayed cerebral ischemia following aneurysmal subarachnoid hemorrhage. Neurosurg. Rev..

[32] Murakami K., Koide M., Dumont T.M., Russell S.R., Tranmer B.I., Wellman G.C. (2011). Subarachnoid hemorrhage induces gliosis and increased expression of the pro-inflammatory cytokine high mobility group box 1 protein. Transl. Stroke Res..

[33] Sun Q., Wu W., Hu Y.C., Li H., Zhang D., Li S., Li W., Li W.D., Ma B., Zhu J.H. (2014). Early release of high-mobility group box 1 (HMGB1) from neurons in experimental subarachnoid hemorrhage in vivo and in vitro. J. Neuroinflamm..

[34] Muhammad S., Chaudhry S.R., Kahlert U.D., Lehecka M., Korja M., Niemelä M., Hänggi D. (2020). Targeting high mobility group box 1 in subarachnoid hemorrhage: A systematic review. Int. J. Mol. Sci..

[35] Zhang X.-H., Peng L., Zhang J., Dong Y.-P., Wang C.-J., Liu C., Xia D.-Y., Zhang X.-S. (2020). Berberine ameliorates subarachnoid hemorrhage injury via induction of sirtuin 1 and inhibiting HMGB1/Nf-κB pathway. Front. Pharmacol..

[36] Haruma J., Teshigawara K., Hishikawa T., Wang D., Liu K., Wake H., Mori S., Takahashi H.K., Sugiu K., Nishibori M. (2016). Anti-high mobility group box-1 (HMGB1) antibody attenuates delayed cerebral vasospasm and brain injury after subarachnoid hemorrhage in rats. Sci. Rep..

[37] Chen T., Pan H., Li J., Xu H., Jin H., Qian C., Yan F., Chen J., Wang C., Chen J. (2018). Inhibiting of RIPK3 attenuates early brain injury following subarachnoid hemorrhage: Possibly through alleviating necroptosis. Biomed. Pharmacother..

[38] Xiong L., Sun L., Zhang Y., Peng J., Yan J., Liu X. (2020). Exosomes from bone marrow mesenchymal stem cells can alleviate early brain injury after subarachnoid hemorrhage through miRNA129-5p-HMGB1 pathway. Stem Cells Dev..

[39] Wang K.-C., Tang S.-C., Lee J.-E., Li Y.-I., Huang Y.-S., Yang W.-S., Jeng J.-S., Arumugam T.V., Tu Y.-K. (2017). Cerebrospinal fluid high mobility group box 1 is associated with neuronal death in subarachnoid hemorrhage. J. Cereb. Blood Flow Metab..

[40] Xing C., Hayakawa K., Lok J., Arai K., Lo E.H. (2012). Injury and repair in the neurovascular unit. Neurol. Res..

[41] Mocchetti I., Wrathall J.R. (1995). Neurotrophic factors in central nervous system trauma. J. Neurotrauma.

[42] Tian X., Sun L., Feng D., Sun Q., Dou Y., Liu C., Zhou F., Li H., Shen H., Wang Z. (2017). HMGB1 promotes neurovascular remodeling via rage in the late phase of subarachnoid hemorrhage. Brain Res..

[43] Schlueter C., Weber H., Meyer B., Rogalla P., Röser K., Hauke S., Bullerdiek J. (2005). Angiogenetic signaling through hypoxia: HMGB1: An angiogenetic switch molecule. Am. J. Pathol..

[44] Lei C., Wu B., Cao T., Zhang S., Liu M. (2015). Activation of the high-mobility group box 1 protein-receptor for advanced glycation end-products signaling pathway in rats during neurogenesis after intracerebral hemorrhage. Stroke.

[45] Jarocka-Karpowicz I., Syta-Krzyżanowska A., Kochanowicz J., Mariak Z.D. (2020). Clinical prognosis for SAH consistent with redox imbalance and lipid peroxidation. Molecules.

[46] Wu F., Liu Z., Li G., Zhou L., Huang K., Wu Z., Zhan R., Shen J. (2021). Inflammation and oxidative stress: Potential targets for improving prognosis after subarachnoid hemorrhage. Front. Cell Neurosci..

[47] Zheng Z.V., Lyu H., Lam S.Y.E., Lam P.K., Poon W.S., Wong G.K.C. (2020). The dynamics of microglial polarization reveal the resident neuroinflammatory responses after subarachnoid hemorrhage. Transl. Stroke Res..

[48] Rojo A.I., McBean G., Cindric M., Egea J., López M.G., Rada P., Zarkovic N., Cuadrado A. (2014). Redox control of microglial function: Molecular mechanisms and functional significance. Antioxid. Redox Signal..

[49] Janko C., Filipović M., Munoz L.E., Schorn C., Schett G., Ivanović-Burmazović I., Herrmann M. (2014). Redox modulation of HMGB1-related signaling. Antioxid. Redox Signal..

[50] Frank M.G., Weber M.D., Fonken L.K., Hershman S.A., Watkins L.R., Maier S.F. (2016). The redox state of the alarmin HMGB1 is a pivotal factor in neuroinflammatory and microglial priming: A role for the NLRP3 inflammasome. Brain Behav. Immunol..

[51] Ved R., Sharouf F., Harari B., Muzaffar M., Manivannan S., Ormonde C., Gray W.P., Zaben M. (2021). Disulfide HMGB1 acts via TLR2/4 receptors to reduce the numbers of oligodendrocyte progenitor cells after traumatic injury in vitro. Sci. Rep..

[52] Yoo H., Im Y., Ko R.-E., Lee J.Y., Park J., Jeon K. (2021). Association of plasma level of high-mobility group box-1 with necroptosis and sepsis outcomes. Sci. Rep..

[53] Yao H.C., Zhao A.P., Han Q.F., Wu L., Yao D.K., Wang L.X. (2013). Correlation between serum high-mobility group box-1 levels and high-sensitivity C-reactive protein and troponin I in patients with coronary artery disease. Exp. Ther. Med..

[54] Sørensen M.V., Pedersen S., Møgelvang R., Skov-Jensen J., Flyvbjerg A. (2011). Plasma high-mobility group box 1 levels predict mortality after ST-segment elevation myocardial infarction. JACC Cardiovasc. Interv..

[55] Angus D.C., Yang L., Kong L., Kellum J.A., Delude R.L., Tracey K.J., Weissfeld L. (2007). Circulating high-mobility group box 1 (HMGB1) concentrations are elevated in both uncomplicated pneumonia and pneumonia with severe sepsis. Crit. Care Med..

[56] Bruchfeld A., Qureshi A.R., Lindholm B., Barany P., Yang L., Stenvinkel P., Tracey K.J. (2008). High mobility group box protein-1 correlates with renal function in chronic kidney disease (CKD). Mol. Med..

[57] Xu D., Zhang Y., Zhang Z.-Y., Tang X.-M. (2021). Association between high mobility group box 1 protein and juvenile idiopathic arthritis: A prospective longitudinal study. Pediatric Rheumatol..

[58] Abdulahad D.A., Westra J., Bijzet J., Limburg P.C., Kallenberg C.G.M., Bijl M. (2011). High mobility group box 1 (HMGB1) and anti-HMGB1 antibodies and their relation to disease characteristics in systemic lupus erythematosus. Arthritis Res. Ther..

[59] Marjanac I., Lovrić R., Barbić J. (2019). Serum levels of the high-mobility group box 1 protein (HMGB1) in children with type 1 diabetes mellitus: Case-control study. Cent.-Eur. J. Immunol..

[60] Wang H., Qu H., Deng H. (2015). Plasma HMGB-1 levels in subjects with obesity and type 2 diabetes: A cross-sectional study in China. PLoS ONE.

[61] Kan M., Song L., Zhang X., Zhang J., Fang P. (2019). Circulating high mobility group box-1 and toll-like receptor 4 expressions increase the risk and severity of epilepsy. Braz. J. Med. Biol. Res. Rev. Bras. Pesqui. Med. Biol..

[62] Festoff B.W., Sajja R.K., van Dreden P., Cucullo L. (2016). HMGB1 and thrombin mediate the blood-brain barrier dysfunction acting as biomarkers of neuroinflammation and progression to neurodegeneration in Alzheimer’s disease. J. Neuroinflamm..

[63] Yang Y., Han C., Guo L., Guan Q. (2018). High expression of the HMGB1-TLR4 axis and its downstream signaling factors in patients with Parkinson’s disease and the relationship of pathological staging. Brain Behav..

[64] Sternberg Z., Sternberg D., Chichelli T., Drake A., Patel N., Kolb C., Chadha K., Yu J., Hojnacki D. (2016). High-mobility group box 1 in multiple sclerosis. Immunol. Res..

[65] Tsukagawa T., Katsumata R., Fujita M., Yasui K., Akhoon C., Ono K., Dohi K., Aruga T. (2017). Elevated serum high-mobility group box-1 protein level is associated with poor functional outcome in ischemic stroke. J. Stroke Cerebrovasc. Dis..

[66] Zhou Y., Xiong K.L., Lin S., Zhong Q., Lu F.L., Liang H., Li J.C., Wang J.Z., Yang Q.W. (2010). Elevation of high-mobility group protein box-1 in serum correlates with severity of acute intracerebral hemorrhage. Mediat. Inflamm..

